# Swimming Exercise Alleviated Insulin Resistance by Regulating Tripartite Motif Family Protein 72 Expression and AKT Signal Pathway in Sprague-Dawley Rats Fed with High-Fat Diet

**DOI:** 10.1155/2016/1564386

**Published:** 2016-10-24

**Authors:** Jie Qi, Bo Yang, Cailing Ren, Jian Fu, Jun Zhang

**Affiliations:** ^1^College of Physical Education, Shanghai Normal University, Shanghai 200234, China; ^2^Department of Food Science and Nutrition, Zhejiang University, Hangzhou 310053, China; ^3^Rehabilitation College of Gannan Medical University, Jiangxi 341000, China; ^4^College of Physical Education, Yangzhou University, Jiangsu 225009, China

## Abstract

We aimed to investigate whether swimming exercise could improve insulin resistance (IR) by regulating tripartite motif family protein 72 (TRIM72) expression and AKT signal pathway in rats fed with high-fat diet. Five-week-old rats were classified into 3 groups: standard diet as control (CON), high-fat diet (HFD), and HFD plus swimming exercise (Ex-HFD). After 8 weeks, glucose infusion rate (GIR), markers of oxidative stress, mRNA and protein expression of TRIM72, protein of IRS, p-AKT^Ser473^, and AKT were determined in quadriceps muscles. Compared with HFD, the GIR, muscle SOD, and GSH-Px were significantly increased (*p* < 0.05, resp.), whereas muscle MDA and 8-OHdG levels were significantly decreased (*p* < 0.05 and *p* < 0.01) in Ex-HFD. Expression levels of TRIM72 mRNA and protein in muscles were significantly reduced (*p* < 0.05 and *p* < 0.01), whereas protein expression levels of IRS-1, p-AKT^Ser473^, and AKT were significantly increased in Ex-HFD compared with HFD (*p* < 0.01, *p* < 0.01, and *p* < 0.05). These results suggest that an 8-week swimming exercise improves HFD-induced insulin resistance maybe through a reduction of TRIM72 in skeletal muscle and enhancement of AKT signal transduction.

## 1. Introduction

Insulin resistance (IR) is the important pathophysiological basis of diseases such as type 2 diabetes mellitus (T2DM), hypertension, hyperlipidemia, and obesity [1, 2]. Insulin primarily acts on liver and fatty tissues, as well as skeletal muscles. Skeletal muscle, which is responsible for more than 30% of energy consumption, is the major peripheral tissue of glucolipid metabolism under insulin stimulation [3]. Several cytokines and peptides, also called myokines, can be synthesized by skeletal muscle cells in type 2 diabetes and are possibly involved in IR [4]. Previously, tripartite motif family protein 72 (TRIM72), also named MG53, is the one with three conserved domains known as “molecular bandage” for muscle cell damage [5–7], while recent studies [8, 9] found that TRIM72 expression is obviously upregulated in pathogenesis of IR and acts as an E3 ligase targeting the insulin receptor and insulin receptor substrate-1 (IRS-1) for ubiquitin-dependent degradation, subsequently inhibiting PI3-K/AKT signal transduction. During IR pathogenesis, abnormally high TRIM72 expression in skeletal muscles was higher than that in other organs, which was the important originating mechanism leading to systemic metabolic disorders [10–12]; it suggests that TRIM72 may be a specific muscle factor in early stage of IR pathological genesis. Moreover, TRIM72 expression is increased under the condition of oxidative stress induced with high-fat diet [5]. These findings suggest that regulation of TRIM72 to AKT signal pathway is involved in the IR pathogenesis.

The insulin receptor substrate (IRS) family of proteins plays a central role in insulin signal transduction. Following activation of the insulin receptor kinase, IRS-1, IRS-2, IRS-3, and IRS-4 undergo phosphorylation of multiple tyrosine residues in unique sequence motifs throughout the molecule. As the studies shown mice with targeted disruption of the IRS genes lend some support to both situations. Despite the absence of IRS-1 and IRS-2, which are the major IRS expressed in all tissues could induce IR, IRS-1 and IRS-2 produce different phenotypes [13–18]; this suggests that IRS-1 and IRS-2 play significant and nonredundant roles in growth and regulation of glucose homeostasis. Studies also demonstrated that IRS-3 and IRS-4 show a restricted tissue distribution and are not critically involved in regulating growth and glucose homeostasis [19–21]. Although studies showed that TRIM72 was targeting IRS-1, whether expression of IRS-2, IRS-3, and IRS-4 is also involved in the TRIM72 in IR rats fed with high-fat diet should need further studies.

**Figure 1:**
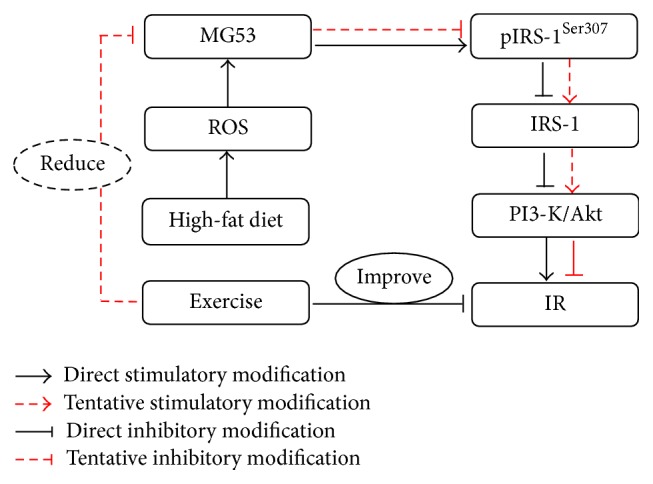
The signaling pathway of high-fat diet induced IR through TRIM72 and PI3-K/AKT in skeletal muscle. Whether the swimming exercise can improve insulin resistance in Sprague-Dawley rats fed with high-fat diet through inhibiting TRIM72 expression and activating PI3-K/AKT signal pathway should be further testified.

Convincing evidences demonstrate that exercise can improve IR [22, 23] partially through PI3-K/AKT signal pathway [24, 25]. However, the detailed mechanisms involved remain to be verified. We therefore established the IR model in Sprague-Dawley rats fed with high-fat diet to observe the effect of swimming exercise on parameters such as lipid deposition, oxidative stress, and TRIM72 expression in the skeletal muscles of the IR rats (see Figure 1). Moreover, we aimed to reveal the possible rule of TRIM72 in IR prevention. We hypothesized that IR can be effectively improved by exercise through regulating TRIM72/IRS-1/AKT pathway and that the decreased TRIM72 may be mediated by the lower levels of ROS which are induced by exercise.

## 2. Material and Methods

### 2.1. Animals

Male Sprague-Dawley rats, aged 5 weeks old with body weight of 130 ± 20 g, provided by the experimental animal center of Jiangsu University (certification number SCXK (Su) 2009-0002), were housed under standard conditions (22 ± 2°C, humidity 50 ± 10%, cycles of 12 h light/12 h dark). Experimental procedures were performed in accordance with the Guidance Suggestions for the Care and Use of Laboratory Animals, formulated by the Ministry of Science and Technology of the People's Republic of China in 1998, and were approved by the Animal Ethics Committee of Shanghai Normal University. Thirty Sprague-Dawley rats were randomly divided into 3 groups: standard diet as control (CON), high-fat diet (HFD), and HFD plus swimming exercise (Ex-HFD). The control diet contained 65% carbohydrates, 22% protein, and 13% fat and the high-fat diet consisted of 40% carbohydrates, 22% protein, and 38% fat.

### 2.2. Exercise Program

The modifications of exercise program were conducted in accordance with the Bendford program [26]. In brief, rats in Ex-HFD group were trained to swim 10–30 min per session for 5 days to reduce water-induced stress. Rats were placed in a plastic pool of 150 × 60 × 70 cm^3^ with a water temperature at 30–32°C; the rats underwent swimming exercise for 45 min twice/day, 6 days/week, for 8 weeks.

### 2.3. Blood Samples Collection and Blood Biochemistry

Twenty hours after the final session of swimming exercise, all rats were anesthetized by single-dose intraperitoneal injection of 10% chloral hydrate (50 mg/kg). Blood samples were collected from abdominal aorta and serum was separated by centrifugation at 1200x rpm for 10 min. Serum glucose level was measured using an autoanalyzer (RT-1904C; Rayto, China). Insulin concentration in serum was determined using a radioimmunoassay described by Laemmli [27].

### 2.4. Calculation of Homeostasis Model Assessment of Insulin Resistance (HOMA-IR)

The HOMA-IR was calculated using the following formula: FBG (mmol/L) × FIN (mIU/L)/22.5 [28], where FBG is fasting blood glucose and FINS is fasting insulin concentration.

### 2.5. Euglycemic Clamp Test

The improved Kraegen method [29] was used to measure the glucose infusion rate (GIR). Briefly, the rats were injected with 50 mg/kg anesthesia containing 10% chloral hydrate and then fixed in supine position. A thin silicon tube was inserted into the jugular vein to connect with peristaltic pump for infusion of insulin and peristaltic pump for infusion of 10% glucose through a three-way tube. Insulin at 4 mU·kg^−1^·min^−1^ and 10% glucose were infused through the bilateral veins (constant speed). Blood sample was collected every 10 min through the venule to measure the blood glucose level, and GIR was constantly adjusted until glucose homeostasis was achieved. Six GIR values were recorded within 60 min under glucose homeostasis, and the average value was used as the GIR under glucose homeostasis.

### 2.6. Skeletal Muscle Sampling

After injecting peritoneal anesthesia with 10% chloral hydrate for 50 mg/kg, the red quadriceps femoris of the bilateral thighs was isolated, placed into liquid nitrogen immediately, and stored at −80°C. Subsequently, 0.9% normal saline was added to the skeletal muscle tissue with a ratio of 50 mg : 50 *μ*L (quality fraction: 10%) to prepare a tissue homogenate by using an ultrasonic crasher (40 amp, 5 sec each time, 10 sec for each interval; operation was repeated five times). The homogenate was centrifuged at 3000 rpm for 15 min under 4°C. Supernatant was collected and stored at −20°C for further testing the level of skeletal muscle TG and oxidative stress parameters.

### 2.7. Measurement of Free Fatty Acid (FFA), Triglyceride (TG), and Oxidative Stress Parameters in Skeletal Muscle

The levels of FFA, TG, SOD, GSH-Px, and MDA in skeletal muscle were determined with free fatty acid determination kit (Sigma), triglyceride determination kit (Sigma), superoxide dismutase determination kit (Sigma), and glutathione peroxidase determination kit (Sigma), respectively, following the manufacturer's instructions. The 8-hydroxyl-deoxyguanosine (8-OHdG) content was measured using enzyme-linked immunosorbent assay (ELISA) (R&D, Minneapolis, USA).

### 2.8. Quantitative Real-Time PCR Analysis

Total RNA was extracted and purified from the cells using the RNA isolator Total RNA Extraction Reagent (TaKaRa, Kusatsu, Japan) and subjected to reverse transcription using the PrimeScript™ RT Master Mix Kit (TaKaRa, Kusatsu, Japan). The real-time PCR and data collection were subsequently performed as described previously [30] using the AceQ® qPCR SYBR® Green Master Mix kit (TaKaRa, Kusatsu, Japan) on ABI 7500 system (ABI, New York, USA). Primers used for the amplification were as follows: TRIM72-F: 5′-CGAGCAGGACCGCACACTT-3′, TRIM72-R: 5′-CCAGGAACATCCGCATCTT-3′; insulin receptor substrate-1 (IRS-1) F: 5′-GAAGAAGTGGCGGCACAAGT3′, IRS-1 R: 5′-GTCAGGCAGAGGCGGTAGAT-3′; IRS-2 F: 5′-ATACCGCCTATGCCTGTCTG-3′, IRS-2 R: 5′-AGAAGAAGCTGTCCGAGTGG-3′; IRS-3 F: 5′-GCAGAGCAGCAAACATGGTA-3′, IRS-3 R: 5′-GCGAAGATCCAAGACTCAGG-3′; IRS-4 F: 5′-TTGCTGACAGTGCCATTTGC-3′, IRS-4 R: 5′-TGCACTTCTTCCTGCCTAGC-3′; and glyceraldehyde-3-phosphate dehydrogenase (GAPDH) F: 5′-GC ACCGTCAAGGCTGAGAAC-3′, GAPDH R: 5′-TGGTGAAGACGCCAGTGG A-3′. The relative expression levels of the indicated mRNA normalized against GAPDH mRNA were calculated using the 2^−ΔΔCT^ methods.

### 2.9. Protein Extraction and Western Blot

Western blot was performed following standard methods. Briefly, the samples were placed in protein extraction solution (lysis in RIPA) and ultrasonicated at maximum speed at 4°C for 30 s (Sonics, Newtown, USA). The homogenate was centrifuged at 12000 rpm at 4°C for 30 min. After denaturation, the samples were subjected to 10% SDS-PAGE and transferred onto polyvinylidene fluoride membranes (Millipore, Billerica, USA). The membranes were then blocked with 5% nonfat dried milk and incubated with primary antibodies and followed by incubation with horseradish peroxidase- (HRP-) linked secondary antibodies. The antibodies used in the current study were rabbit polyclonal anti-IRS-1 (1 : 2000, Cell Signaling Technology, MA, USA), rabbit polyclonal anti-pIRS-1 (Ser307) (1 : 2000), rabbit polyclonal anti-IRS-2 (1 : 1000, CST), and HRP-linked anti-rabbit IgG (1 : 2000); rabbit polyclonal anti-IRS-3 (1 : 200, Santa Cruz Biotechnology, CA, USA), goat polyclonal anti-IRS-4 (1 : 200), goat polyclonal anti-TRIM72 (1 : 200), and HRP-linked anti-goat IgG (1 : 2000); and mouse monoclonal anti-GAPDH (1 : 1000) and HRP-linked donkey anti-goat IgG (1 : 3000) (KangChen Bio-tech, Shanghai, China). Immunoreactive protein bands were visualized with the Pierce ECL Plus Western Blot Substrate (Thermo Fisher Scientific, Rockford, IL, USA). GAPDH was performed as an internal loading control, and quantification of the band intensity was performed using ImageJ software (NIH) and defined as the fold-change of the CON after being normalized against GAPDH.

### 2.10. Statistical Analysis

Firstly, we test the normality of data distribution; the data with normal distribution were expressed as mean (M) ± standard deviation (SD) and the data with skewed distribution were log-transformed. Pearson correlation analysis was performed for the parameters, and one-way ANOVA for significance was employed for intergroup data. Multiple comparisons were performed using LSD. The level of significant difference was set at *p* < 0.05. All analyses were completed with SPSS (version 17.0, SPSS Inc., 233 S Wacker Drive, 11th Floor, Chicago, IL 60606).

## 3. Results

### 3.1. Blood Glucose, Blood Insulin, and Insulin Resistance Index

**Figure 2:**
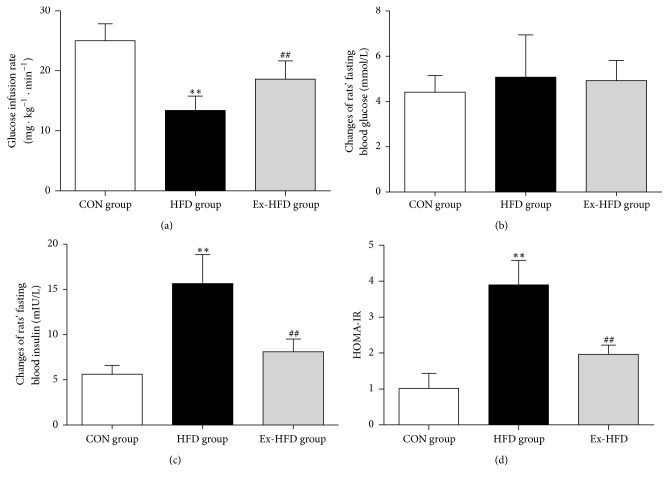
Blood glucose, insulin, and insulin resistance index in the three groups. (a) The fasting blood glucose in three groups at the 8th week. There were no significant differences in the fasting blood glucose measured after 8-week swimming exercise intervention compared with the untreated high-fat-diet group. (b) The fasting blood insulin in three groups at the 8th week. There was significant decrease in the fasting blood insulin that was measured after 8-week swimming exercise intervention compared with the untreated high-fat-diet group. (c) The GIR of three groups. The GIR is commonly used in evaluating insulin resistance. After training with swimming exercise, GIR in group Ex-HFD was significantly increased. (d) HOMA-IR value of three groups at the 8th week, which was also selected to evaluate insulin resistance. After training with swimming exercise, HOMA-IR was improved. ^*∗∗*^ *p* < 0.01, versus CON group; ^##^ *p* < 0.01, versus group HFD group.

To evaluate insulin sensitivity, euglycemic clamp was firstly performed; the results showed that the GIR of group HFD considerably decreased (*p* < 0.01) compared with that in group CON, while the GIR in rats of group Ex-HFD significantly increased (*p* < 0.01) compared with that in group HFD (Figure 2(a)). Furthermore, to assess IR, we next detected fasting blood glucose (FBG) and fasting blood insulin (FIN) to calculate HOMA-IR index. As shown in Figures 2(b), 2(c), and 2(d), there were no significant differences of FBG among group CON, group HFD, and group Ex-HFD (*p* > 0.05, Figure 2(b)); the level of the FIN in group HFD was significantly higher than that in group CON (*p* < 0.01); the FIN in the Ex-HFD group was significantly lower than that in HFD group (*p* < 0.01, Figure 2(c)); and the HOMA-IR was markedly increased (*p* < 0.01) compared with that in group CON. However, the HOMA-IR was greatly decreased (*p* < 0.01) compared with that in group HFD (Figure 2(d)). These results proved that the animal model prepared using high-fat diet is suitable for the current study, and an 8-week swimming exercise can improve IR in rats induced by high-fat diet.

### 3.2. FFA, TG, and Oxidative Stress Parameters in Skeletal Muscles

To observe the lipid accumulation in skeletal muscles after a long-time high-fat diet in rats, we measured the levels of FFA and TG. As shown in Table 1, the FFA and TG of the skeletal muscle in group HFD were significantly higher than those in group CON (*p* < 0.01, resp.). The FFA and TG of the skeletal muscle in group Ex-HFD were significantly lower than those in group HFD (*p* < 0.05). Moreover, to confirm the oxidative stress in skeletal muscles after a long-time high-fat diet in rats, we also detected the levels of SOD, GSH-Px, MDA, and 8-OHdG in rat skeletal muscles. For the test, the levels of SOD and GSH-Px in group HFD were significantly decreased (*p* < 0.05, resp.) than those in group CON, while MDA and 8-OHdG contents of the skeletal muscles in group HFD obviously increased (*p* < 0.05 and *p* < 0.01, resp.). After 8 weeks of exercise, the SOD and GSH-Px activities in group Ex-HFD significantly increased compared with those in group HFD (*p* < 0.05 and *p* < 0.01, resp.). However, the MDA and 8-OHdG contents in group Ex-HFD significantly decreased (*p* < 0.05 and *p* < 0.01, resp.). These findings indicate that 8-week swimming exercise can reduce the lipid accumulation and the level of oxidative stress in skeletal muscles of rats after being fed with high-fat diet.

### 3.3. Quantitative Real-Time PCR Analysis

**Figure 3:**
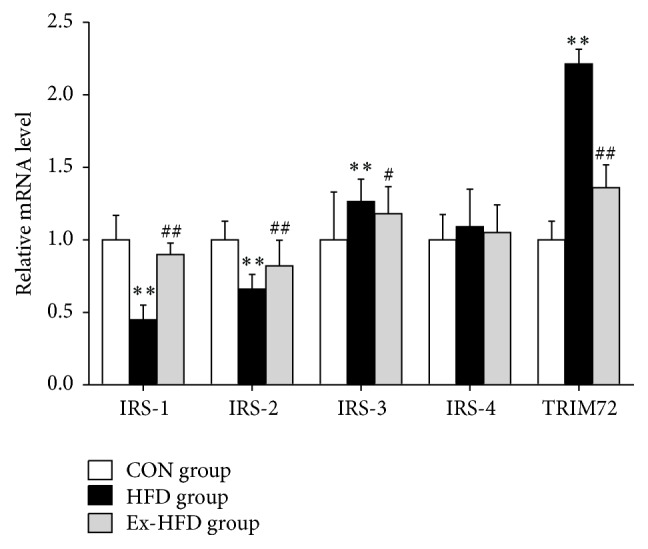
Effect of swimming exercise on mRNA expression of IRS-1 and TRIM72 in skeletal muscle. Data are presented as mean ± SD. For each group *n* = 10. IRS-1: insulin receptor substrate-1, IRS-2: insulin receptor substrate-2, IRS-3: insulin receptor substrate-3, IRS-4: insulin receptor substrate-4, and TRIM72: tripartite motif family protein 72. ^*∗∗*^ *p* < 0.01: versus CON group; ^##^ *p* < 0.01: versus group HFD group; ^#^ *p* < 0.05: versus group HFD group.

Since high-fat diet could induce oxidative stress, we further verified the effects of exercise on TRIM72 and IRS gene expression in skeletal muscle induced by oxidative stress after high-fat diet, and qPCR was performed. As shown in Figure 3, the mRNA expression of TRIM72 and IRS-3 in group HFD was significantly higher than those in group CON (*p* < 0.01 and *p* < 0.01, resp.); mRNA expression of IRS-1 and IRS-2 in group HFD was significantly lower than that in group CON (*p* < 0.01, resp.). Compared with group HFD, the mRNA expression of TRIM72 and IRS-3 in group Ex-HFD significantly decreased (*p* < 0.01 and *p* < 0.05, resp.); the mRNA expression of IRS-1 and IRS-2 in group Ex-HFD was significantly increased than those in group HFD (*p* < 0.01). No significant differences of mRNA expression of IRS-4 were shown among the group CON, group HFD, and group Ex-HFD. These findings demonstrate that exercise can decrease the mRNA expression of TRIM72 and IRS-3 and increase the mRNA expression of IRS-1 and IRS-2 of skeletal muscles in rats fed with high-fat diet.

### 3.4. Western Blot Analysis

**Figure 4:**
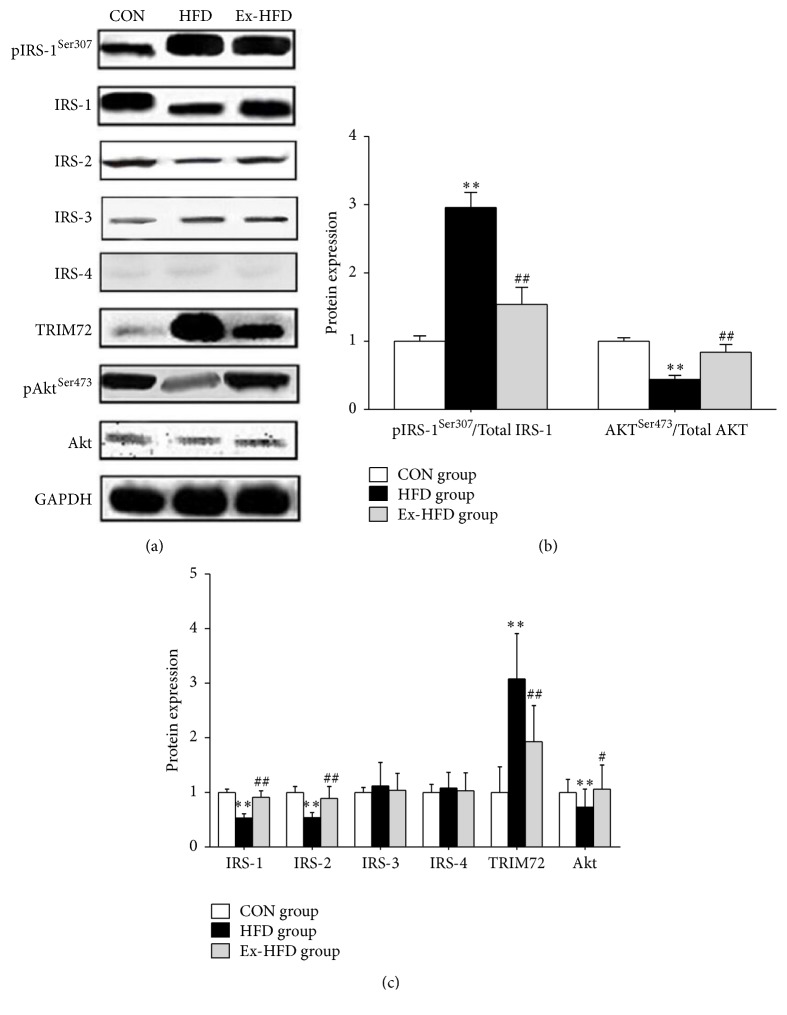
Protein expression of TRIM72, IRS-1, IRS-2, IRS-3, and IRS-4 and phosphorylation of IRS-1 (Ser307) and AKT (Ser473) levels in skeletal muscle were tested by western blot. ^*∗∗*^ *p* < 0.01: versus CON group; ^*∗*^ *p* < 0.05: versus CON group; ^##^ *p* < 0.01: versus group HFD group; and ^#^ *p* < 0.05: versus group HFD group.

TRIM72, pIRS-1^Ser307^, IRS-1, IRS-2, IRS-3, IRS-4, pAKT^Ser473^, and AKT protein expression levels were detected by western blot method in skeletal muscle. To prove the effects of exercise on protein expression of TRIM72, IRS, and AKT in skeletal muscle induced by long-term high-fat diet, we performed western blot. As shown in Figure 4, the TRIM72 protein expression in group HFD was significantly higher than that in group CON (*p* < 0.01); the TRIM72 protein expression in group Ex-HFD significantly decreased compared with that in group HFD (*p* < 0.01). Furthermore, compared with group CON, the phosphorylation level of IRS-1^Ser307^ was significantly increased (*p* < 0.01); the expression level of IRS-1 and IRS-2 protein was significantly decreased in group HFD (*p* < 0.01). After swimming exercise for 8 weeks, compared with group HFD, the phosphorylation level of IRS-1^Ser307^ significantly decreased (*p* < 0.01); the expression level of IRS-1 and IRS-2 protein significantly increased (*p* < 0.01) in group Ex-HFD while the expression of IRS-3 and IRS-4 showed no significance among group CON, group HFD, and group Ex-HFD. In addition, the proteins of pAKT^Ser473^ and AKT in group HFD were significantly lower than those in group CON (*p* < 0.01 and *p* < 0.05, resp.), but significantly increased in group Ex-HFD than those in group HFD (*p* < 0.01 and *p* < 0.05, resp.). These findings suggest that swimming exercise could decrease the protein expression of TRIM72, upregulate the protein expression of IRS-1 and IRS-2, and phosphorylation level of AKT (Ser473) of skeletal muscles in rats fed with high-fat diet.

## 4. Discussion

In present study, we tested the levels of oxidative stress parameters and performed qPCR and western blot to detect the mRNA and protein of TRIM72, IRS (IRS-1, IRS-2, IRS-3, and IRS-4), and AKT and protein of phosphorylation of IRS-1 (Ser307) and AKT (Ser473). The results showed that the elevation of oxidative stress, TRIM72, and pIRS-1^Ser307^ expression and the reduction of protein expression of IRS-1 and pAKT^Ser473^ of skeletal muscle in rats fed with high-fat diet, while the swimming exercise was effective in reversing IR induced by high-fat diet. Our findings indicate that an 8-week of swimming exercise not only effectively reduced the oxidative stress of skeletal muscle, but also alleviated the expression of TRIM72, upregulated the AKT signaling pathway, and reversed IR.

It has been widely accepted that lipid accumulation and oxidative stress are strongly correlated with the IR in peripheral tissues (muscle and liver) [31–37]. Long-term excessive intake of high calories could lead to excessive fat accumulation in fat cells, consequently resulting in fats flowing to other tissues except fat tissues. Ectopic lipid deposition subsequently occurs, thereby causing cell damage. Studies [4, 38] have shown that excessive TG accumulation in nonfat tissue has been known to have a toxic effect on cells and to reduce sensitivity to insulin, eventually leading to diabetes and metabolic syndrome [31, 39]. We fed the rats with high-fat diet, and we noticed that high-fat diet induced the increase in visceral fat content along with fat decomposition, which could lead to massive FFA and TG in the skeletal muscle. The present study also demonstrated that GIR greatly decrease in rats fed with high-fat diet, and the ISI significantly increased simultaneously, while HOMA-IR was obviously decreased, suggesting that the 8-week high-fat diet led to a presence of IR in rats. However, the GIR and ISI were significantly increased in rats from the exercise group that were also fed with high-fat diet, whereas HOMA-IR considerably decreased, implying that IR genesis and progress in rats fed with high-fat diet can be effectively relieved by swimming exercise.

Changes in in vivo metabolism (such as massive lipid accumulation and increased ROS) [10, 40] eventually lead to cell damage. TRIM72, as a specific cell repair factor of skeletal muscle, is rapidly and massively transposed toward the damaged sites [5]. When experiencing a long-term high-fat diet, the oxidative stress could occur, which consequently results in oxidative damage in skeletal muscle cells. Subsequently, TRIM72 expression is initiated. Previous studies have demonstrated that SOD, MDA, GSH-Px, and 8-OHdG can evaluate the oxidative stress [41–45]. Several studies have shown that systemic IR can be effectively improved by exercise through reduction of oxidative stress [46, 47]. In the present study, we tested SOD, MDA, GSH-Px, 8-OHdG, and TRIM72 levels in the skeletal muscles of rats in all the groups; the oxidative stress was found increased after long-term feeding with high-fat diet, and, in HFD group, the TRIM72 showed a significant-level promotion on both mRNA and protein expression; this is in accordance with Song et al. [8] (published in “*Nature*” 2013), although some publications from different groups showed that HFD treatment does not alter expression of TRIM72 [48–53]. We analyzed and inferred the possible reasons: firstly, the differences of the high-fat diet composition: the multiple researchers use the high-fat diet with 45% or more calories from fat or extra cholesterol added. In our study, the high-fat diet only contains 38% calories from fat. Meanwhile, multiple researchers performed feeding mice with high-fat diet for a long time (8 weeks, 16 weeks, or more weeks); secondly, Song et al. showed that TRIM72 expression increased at the early stage of IR, in other words, TRIM72 expression increased when mild IR existed. While the multiple studies fed mice with high-fat diet (45%, 60.9%, or more calories from fat) for long, the data showed the seriously impaired insulin action; it indicated severe IR progression in mice. However, we fed rats with high-fat diet (38% calories from fat) for 8 weeks, the IR was milder than those mice fed with high-fat diet (45%, 60.9%, or more calories from fat); it might be the early stage of IR. Therefore, we observed that TRIM72 expression markedly increased in group HFD, in keeping with Song et al. In addition, we found that, in HFD group, the mRNA and protein expression of IRS-1 and IRS-2 were both notably elevated; the mRNA expression of IRS-3 was greatly increased, while its protein expression showed no significance; the mRNA and protein expression of IRS-4 have not obviously changed. However, these levels can be effectively reduced by an 8-week swimming exercise. In the present study, we also detected the expression of IRS (IRS-1, IRS-2, IRS-3, and IRS-4). Meanwhile, the AKT signal was determined. In the group HFD, pIRS-1^Ser307^, IRS-1, IRS-2, and pAKT^Ser473^ expressions were at a lower level. While 8 weeks of swimming exercise later, these levels of protein were all increased. Therefore, we infer that 8-week high-fat diet can result in high expression of TRIM72 mRNA and protein, and TRIM72 acts as an E3 ligase targeting the insulin receptor and IRS-1 for ubiquitin-dependent degradation, then inhibiting PI3-K/AKT signal transduction and subsequently producing IR. In the study, we found that the level of IRS-2 increasing is not relevant to the pathway that we studied, which may be through other ways.

Previous studies have shown that systemic IR can be effectively improved by exercise through reduction of oxidative stress [46]. In the present experiment, relevant parameters such as expression of TRIM72, IRS, and AKT in skeletal muscles of rats in Ex-HFD groups showed that oxidative stress reduced after long-term exercise with high-fat diet. Subsequently, the expression of TRIM72 mRNA and protein levels was decreased, and insulin signal transduction was enhanced through swimming exercise. These results suggest that an 8-week swimming exercise improves HFD-induced insulin resistance, maybe through a reduction of TRIM72 in skeletal muscle and enhancement of AKT signal transduction.

## 5. Summary

IR induced by high-fat diet can be effectively improved during an 8-week swimming exercise. The possible mechanism in rats fed with high-fat diet during exercise implied that the degree of damage in the skeletal muscle cells can be relieved by the decrease in lipid deposition and inhibition of oxidative stress in skeletal muscles. Consequently, this finding indicates reduced TRIM72 expression level and increased IRS-1 protein expression level can enhance skeletal muscular PI3-K/AKT signal transduction in rat skeletal muscles, then finally improving IR.

## Figures and Tables

**Table 1:** Effect of swimming exercise on FFA, TG, and oxidative stress parameters in skeletal muscle.

	CON	HFD	Ex-HFD
FFA (nmol/g pro)	2.32 ± 0.77	5.59 ± 1.39^*∗∗*^	3.28 ± 0.83^*∗*^
TG (mmol/g pro)	0.35 ± 0.12	0.28 ± 0.09^*∗∗*^	0.38 ± 0.18^#^
SOD (U/g pro)	19.57 ± 6.55	12.09 ± 5.72^*∗*^	18.15 ± 5.15^#^
GSH-Px (U/g pro)	0.84 ± 0.10	0.41 ± 0.09^*∗*^	0.66 ± 0.12^##^
MDA (nmol/g pro)	15.31 ± 2.07	19.34 ± 3.32^*∗*^	15.51 ± 3.72^#^
8-OHdG (ng/g pro)	289.35 ± 19.84	538.72 ± 61.71^*∗∗*^	418.77 ± 42.87^##^

FFA, triglycerides, SOD, GSH-Px, MDA, and 8-OHdG of all rats were measured at the 8th week of the experiment. Results were expressed as mean (M) ± standard deviation (SD) (*n* = 10). Differences between groups were compared by one-way analysis of variance (ANOVA). We could observe an increase in FFA, TG, MDA, and 8-OHdG and a decrease in SOD and GSH-Px in IR rats, but exercise could decrease the levels of FFA, TG, MDA, and 8-OHdG and increase SOD and GSH-Px in skeletal muscle of rats. ^*∗∗*^ *p* < 0.01: versus CON group; ^*∗*^ *p* < 0.05: versus CON group; ^##^ *p* < 0.01: versus group HFD group; and ^#^ *p* < 0.05: versus group HFD group.
